# The worsening divergence of biotechnology: the importance of risk culture

**DOI:** 10.3389/fbioe.2023.1250298

**Published:** 2023-08-30

**Authors:** Benjamin D. Trump, Christopher L. Cummings, Nicholas Loschin, Jeffrey M. Keisler, Emily M. Wells, Igor Linkov

**Affiliations:** ^1^ United States Army Corps of Engineers, Washington, DC, United States; ^2^ Department of Genetic Engineering and Society Center, North Carolina State University, Raleigh, NC, United States; ^3^ Department of Sociology, Iowa State University, Ames, IA, United States; ^4^ Department of Management Science & Info Sys, University of Massachusetts Boston, Boston, MA, United States

**Keywords:** biotechnology, governance, divergence, risk culture, prospect theory

## Abstract

In the last 20 years, the field of biotechnology has made significant progress and attracted substantial investments, leading to different paths of technological modernization among nations. As a result, there is now an international divide in the commercial and intellectual capabilities of biotechnology, and the implications of this divergence are not well understood. This raises important questions about why global actors are motivated to participate in biotechnology modernization, the challenges they face in achieving their goals, and the possible future direction of global biotechnology development. Using the framework of prospect theory, this paper explores the role of risk culture as a fundamental factor contributing to this divergence. It aims to assess the risks and benefits associated with the early adoption of biotechnology and the regulatory frameworks that shape the development and acceptance of biotechnological innovations. By doing so, it provides valuable insights into the future of biotechnology development and its potential impact on the global landscape.

## 1 Introduction

Over the past 2 decades, biotechnology has experienced significant advancements, fueled by large infusions of capital and institutional development. However, this progress has taken place against a backdrop of uncertain social, economic, and risk-based concerns that threaten to derail national technology modernization plans for certain countries or stymie future development altogether. The result is a burgeoning international divergence in commercial and intellectual capabilities, with some nations adopting a slower, more risk averse development pathway while others seek primacy in one or more permutations of biotechnology. The implications of this divergence are unknown, though questions abound regarding what countries might do about it. More directly: why did this divergence arise, why is it worsening, and might future global biotechnology development look like if this trend is unchanged for the next decade?

Addressing these questions requires an understanding of the perceived incentives that global actors have in engaging biotechnology modernization. Such modernization does not happen by accident, requiring hundreds of millions of dollars and a concerted effort to develop the human capital and subsequent market demand to sustain innovation upon the conclusion of initial government investment. The overall requirement to reach this end-stage is that any commercial-ready materials or composite products possess relatively well-understood risk profiles consistent with domestic requirements and norms, while the characteristics and behavior of such materials is predictable under recommended circumstances.

Unfortunately, reaching these desired endpoints is an uncertain process fraught with many challenges. Overcoming the technical rate-limiting steps that enable scientific progress is not guaranteed. As such, any potential benefits accrued by unlocking a technology’s capabilities must be discounted by the potential for failure throughout the development process, as well as any institutional, social, economic, or security concerns that various stakeholders would have in approving of and supporting technology modernization. The risk, governance, and ELSI (ethnical, legal, and social implications) surrounding biotechnology worsen this discounting factor—presenting considerable hurdles that many nations would face in their modernization process. The result of 2 decades of biotechnology development has resulted in a wide and broadening gulf between countries with interest and capability in biotechnology modernization that is likely to worsen without corrective action over the next 20 years.

## 2 Why does technology divergence happen?

At face value, emerging technologies promise benefits that present societies lack, and offer improvements to quality of living. Often, these improvements are iterative—a refinement or increased efficiency to a current process or capability. Occasionally, the improvements are revolutionary—posing benefits that have little to no corollary within current markets or technological capacities. Generally, evolutionary benefits (e.g., improving crop yield and nutritional value) carry less technical risk and are more likely to succeed, though produce less net societal value than revolutionary benefits (novel treatment for a debilitating illness).

If deemed of interest, developers and governments seek both types of benefits as “early adopters”. Defined as actors who invest in the earliest years of a technology’s development, and prior to the introduction of marketable products, early adopters reap the benefits of being at the forefront of innovation. These benefits can be multifaceted, including economic gains from commercialization, the prestige of technological leadership, the strategic advantage of possessing proprietary knowledge, and the societal benefits of improved services and products. Importantly, these benefits often influence the trajectory of technology development, with developers and governments strategically investing in areas they believe will yield the highest return on investment.

The early adopter dynamic can also create a feedback loop, where the countries that are the most successful in developing and adopting new biotechnologies attract more investment, talent, and political support for future biotech endeavors. This is particularly true for technologies requiring a massive up-front cost with few barriers to maturation, such as advanced rocketry and the space race, to cases where the ability to innovate is tightly controlled, contested, and of a military nature (e.g., competition for nuclear energy and the atomic bomb in the 1940s and 1950s). Ultimately, the ability of an early adopter to successfully innovate and capture portions of a new market contributes to a self-reinforcing cycle of technology leadership, capturing a greater portion of potential technological and economic benefits from innovation, as well as shaping the global trade and regulatory system to be more in-line with the norms, values, and modernization objectives of early adopter nations.

However, being an early adopter of technology is not without risks. The trajectory of technological progress is notoriously difficult to predict, with a high degree of uncertainty surrounding both the technical feasibility of emerging technologies and the societal response to these technologies. Early adopters must navigate this uncertainty, balancing the potential rewards of successful innovation against the risks of technological failure, public backlash, or unintended consequences.

It is because of these risks that many nations opt for a more risk-averse approach to innovation—particularly when the technology in question or its potential applications clash with local institutions, regulatory instruments, as well as domestic ELSI norms and values. Such hesitancy to innovate in certain areas may persist despite enormous potential benefits, following the precepts of prospect theory on a societal scale (Kahneman and Tversky, 2012). They prioritize the management of potential risks and the prevention of harm over the pursuit of potential benefits and/or the considerable expense of funding technically uncertain scientific endeavors. The regulatory frameworks in these countries often embody the precautionary principle, requiring extensive evidence of safety and efficacy before new technologies can be approved. This approach can slow the pace of technology development and adoption but is seen by these nations as a necessary trade-off to protect public health, the environment, and societal values in the short to intermediate term.

Applications of prospect theory, rooted in behavioral economics and psychology, have gained significant attention in the field of international governance and policy comparisons. Its conceptual framework provides a lens through which to examine decision-making processes and outcomes at both individual and national levels. Traditional risk evaluation methods are prescriptive, such as with guiding biotechnological developments in a manner congruent with rationality and objective economic tradeoffs. However, prospect theory serves as a descriptive counterpoint, acknowledging the reality that decision-making, whether in the pursuit of biotechnological advancements or the formation of governing policies, is not always aligned with predicted rational outcomes. The theory underscores how cognitive biases, such as framing, can lead stakeholders away from objectively beneficial choices in biotechnology—particularly in an environment of heightened uncertainty relative to technology hazards, exposure pathways, and health consequences. Acknowledging these biases offers an opportunity for intervention, enabling the creation of strategies that consider and counteract these biases.

Since Kahneman and Tversky’s initial discussion of prospect theory, its applications for international risk governance have entered into various applications. For instance, Mercer (2005) reviewed and applied prospect theory to the field of political science to evaluate decision-making under conditions of uncertainty and policy choices in the realm of international politics. By extension, Levy (1996) employed prospect theory to evaluate governance issues including who conducted a two-level analysis to investigate the interaction between the individual-level prospect theory and the systemic-level security dilemma to evaluate how political leaders of adversarial states behave differently when they are bargaining over gains than when they are bargaining over losses. More recently, Ross (2020) applied prospect theory to evolving standard operating procedures and decision-making in Afghanistan and explored how psychological biases influence risk calculation and decision-making, emphasizing the significance of reference points and how they are modulated. Further applications of prospect theory have evaluated competing strategic behaviors with regard transjurisdictional water pollution (Yuan et al., 2022) emergency decision-making regarding water diversion in China (Li et al., 2022) and blockchain-based data governance and government policy incentives for manufacturing supply chains (Wei et al., 2023).

**FIGURE 1:**
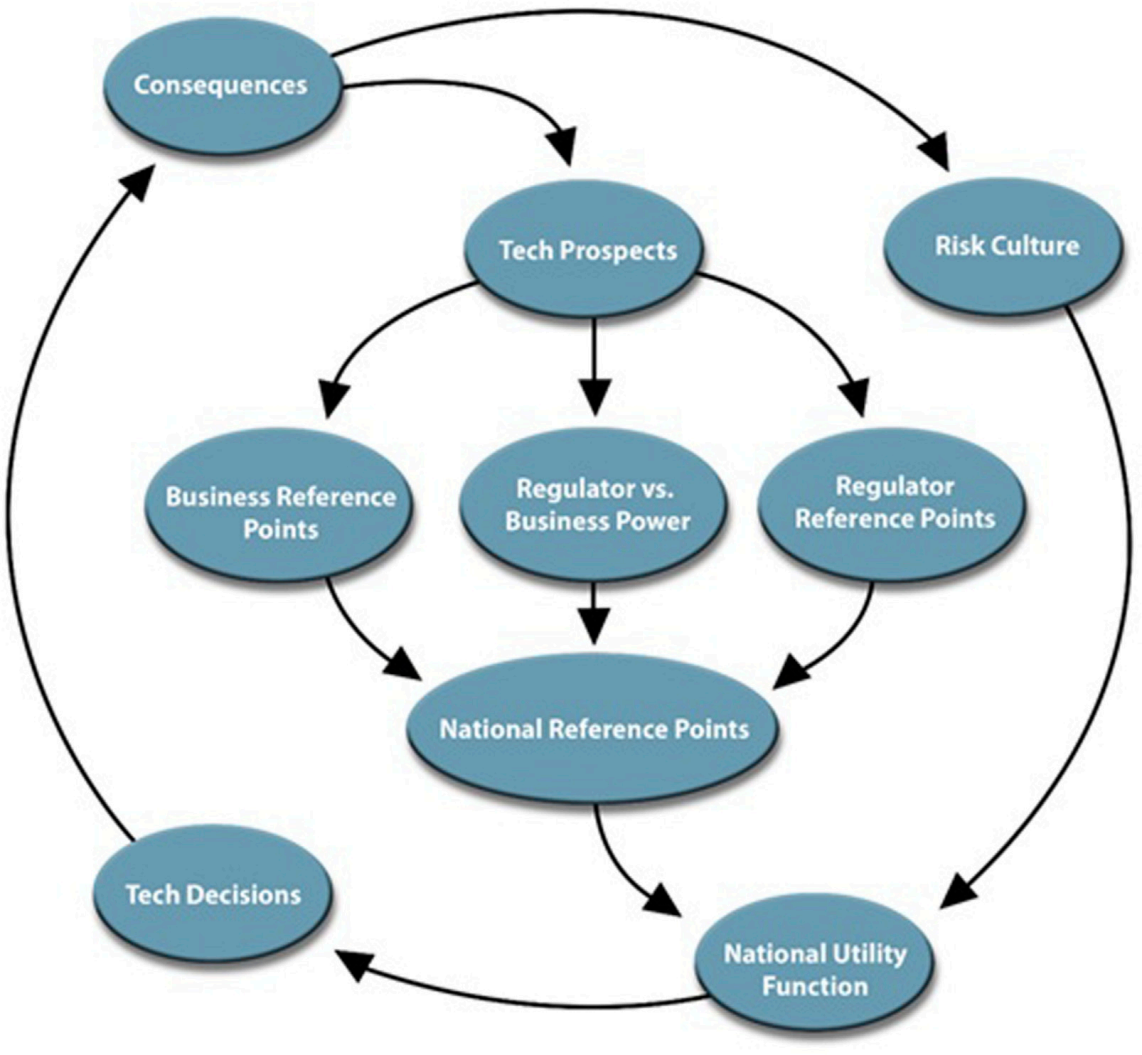
Flow diagram illustrating international competition for technology innovation based upon technology prospects and risk culture.

From this foundation, we identify that, for biotechnology, prospect theory may best inform the institutional, political, and social values and constraints that frame innovation tradeoffs for countries inform national “risk culture” that, alongside the perceived prospects of a given innovation, inform national desire to engage in a potentially risky technology modernization endeavor (Figure 1). During the earliest stages of technology development, the capabilities and products resulting from potential technology maturation are assessed based upon stated political and institutional goals, as well as desired needs for economic competitiveness, national defense, and overall societal wellbeing (Corona et al., 2006; Titus et al., 2020; Zhang, 2020). In turn, these prospects are evaluated through different regulatory and industry frames, balancing potential returns on technology investment against direct or indirect human and environmental health hazards. These frames, alongside social perceptions and demand for technology innovation, form the impetus of technology modernization platforms that inevitably inform policy (Jasanoff, 1987).

National risk culture is a pervasive influence on both top-down and bottom-up governance, ranging from how regulators and legislators perceive risk of an emerging technology, to the willingness of the public and markets to embrace new technologies, their products, and the benefits associated with marketable innovation (Jasanoff, 2015). Top-down, regulators informed by a risk-averse culture may seek to impose stringent controls on the development and deployment of new technologies, demanding high levels of evidence of safety and efficacy, and prioritizing the avoidance of potential harm. On the other hand, in a risk-tolerant culture, regulators may be more inclined to adopt a flexible approach, allowing innovation to proceed with appropriate oversight, while continuously monitoring and adjusting regulatory measures in response to new information about risks and benefits. These frames are difficult to change outside of a focusing event, such as a major technology breakthrough, or an international accord (e.g., the Cartagena Protocol on Biosafety) (Lee and Malerba, 2017).

From the bottom-up, the public and market’s acceptance of new technologies is also shaped by the prevailing risk culture. Public trust in government institutions is crucial; faith in the government’s ability to regulate and monitor new technologies with uncertain and potentially hazardous properties can significantly alter public and market acceptance. Prevailing ethics and cultural values also play a pivotal role. For instance, societies prioritizing environmental sustainability might be more accepting of innovations in green technologies, despite potential risks, than those where economic growth is prioritized above all else. For biotechnology, perceived trustworthiness of policymakers informs social and market enthusiasm for technology modernization efforts. A demonstrative case includes China, which in the aftermath of the “CRISPR-baby scandal”, revised hard law codes via the Chinese Ministry of Justice as well as the Ministry Science and Technology established clearer requirements for the handling of human genetic resources (State Council of China, 2019; Araz, 2020). Simultaneously in July 2019, China established the National Science and Technology Ethics Committee to address ELSI concerns of various emerging technologies (Araz, 2020). All regulatory and policy developments exist against a greater backdrop of a dedicated drive for field leadership of biotechnology in the life sciences, including over $100 billion in public funding that has been invested into Chinese biotechnology research, particularly on the life sciences (Moore, 2020). The stringency of ethical and legal proscriptions is debated (Araz, 2020), though the improved *de jure* policy structure alongside substantial financial incentives push Chinese advancement in an area of biotechnology research with heightened risks, and competition against western nations with stringent controls and public skepticism of human subjects research (Akin et al., 2017).

In risk-averse societies, consumers may be skeptical of products derived from new technologies, demanding transparent information about their development and potential risks. This consumer skepticism can influence market dynamics, potentially discouraging investment in innovative but risk-associated technologies. Conversely, in societies with a risk-tolerant culture, there may be greater public and market enthusiasm for new technologies, driving investment and rapid adoption of innovative products. Thus, national risk culture, acting from both top-down and bottom-up, can significantly influence the pace and direction of technology modernization in a country based upon discounted evaluations of likely short-term risks against potential longer-term early adopter benefits.

Technology divergence occurs when risk culture becomes increasingly entrenched for a given innovation, and the potential benefits of the innovation are not perceived as revolutionary enough to contravene regulatory practice and societal expectations (Liebowitz and Margolis, 1995). Once an innovation is tagged as being excessively risky within a certain risk culture, it becomes difficult to reverse this perception, even with emerging evidence of safety or efficacy. One example includes perceptions of engineered agriculture in the European Union, which throughout decades of research and commodification, still encounters both public and regulatory reluctance to approve the importation, planting, and consumption of genetically modified crops that can be traced back to early concerns of GMO safety in the 1990s (Jiang, 2020). Likewise, the pursuit of early actor privileges places increased political and market pressure on successfully translating innovation to markets—even if potential hazards are not fully characterized, exposure pathways are less than certain, and consequences are questionable. Unless a tremendous shift occurs to stimulate development (e.g., the successful launch of Sputnik that ignited the space race) or limit marketability (e.g., the Chernobyl and Fukushima Daiichi nuclear catastrophes), nations are likely to continue on their existing risk culture pathways until interest in the innovation fizzles out, or it switches from an “emerging” to an “emerged” technology, with established markets, safety and security norms, and general best practices and operating procedures.

This process can take years or decades, with the implications of uneven international technology development uncertain until risk culture entrenchment is well underway. Even then, as a technology matures, differences in technology framing can drive practitioners, regulators, and civil societies from different nations to interpret the “winners and losers” of the innovation race differently and create self-fulfilling prophecies. Risk averse nations can point to instances of safety or security breakdown as proof that their wariness of rapid innovation is justified, while early adopters frame their economic, technical, and social benefits from marketable innovation as proof of how aggressive innovation can lift standards of living. While identifying true winners and losers is often difficult, cases emerge where the aggressive innovator is unable to overcome technical or safety hurdles prior to marketability, or when risk averse nations become reliant upon early adopters for some critical and high-demand benefit for their businesses, consumers, and citizens. Both outcomes carry tremendous strategic risk for economic competitiveness and national security.

## 3 Why is technology divergence worsening for biotechnology?

Biotechnology’s progress is marked by a particularly contentious debate that fuels self-reinforcing technology divergence. Simultaneously, emerging biotechnologies like synthetic biology possess unknown, potentially extreme, and possibly irreversible risks (e.g., gene transfer, introduction of invasive species that disrupts local ecosystems, unforeseen harms to human subjects, potential self-sustaining persistence in the environment), while also must contend with decades of difficult debates regarding the safety, security, ethics, and benefits of early research into genetic engineering due to breakthroughs in understanding of DNA and its synthesis (Berg et al., 1975; Barkstrom, 1985; Abels, 2005; Hurlbut, 2015; Parthasarathy, 2015; Bier, 2022). Heated historical debates formed the battlelines by which much of present-day biotechnology and synthetic biology are waged, while the novelties of emerging research foster an even broader risk-reward gamble for countries considering biotechnology modernization.

Historical breakthroughs with significant debate on the future of biotechnology governance included the creation of the first transgenic animal in 1985 (pigs), as well as transgenic corn in 1988 (Klein et al., 1988). These advancements eventually contributed to the rise of genetically modified organisms being sold in markets, such as with the engineered tomato in 1994 (Uzogara, 2000). The development and maturation of genetic engineering during this period focused on addition, deletion, or substitution of specific DNA base pairs, where more substantial genetic modification of cellular systems was limited by technological constraints of the day (Cameron et al., 2014). Debates raged through this early period regarding safety concerns and good governance challenges of engineered products (Kuzma, 2022), as well as the dual-use nature of many biotechnology breakthroughs (Epstein, 2005), their potential misuse (Mueller, 2019), and broader ethical challenges by skeptical publics ranging from religious (Austriaco, 2020), to moral (Midgley, 2000), to commercial (Mitalipov, 2017), to personal preferences for less exposure to genetically engineered organisms (GMOs) (Sagar et al., 2000).

More recently, advancements in automated DNA sequencing, coupled with sophisticated computational tools, have enabled high-throughput methodologies to analyze RNA, proteins, lipids, and metabolites and create extensive libraries of cellular components (Cameron et al., 2014). This step up in genetic research, coupled with continued reduction in cost of genetic sequencing and synthesis, have facilitated a systems engineering approach to biology. From the early 2000s and onward, genetic engineers pondering questions of whether complex cellular networks could be viewed as an engineered system, where deliberate biological engineering of a cell’s DNA could yield complex changes to how those systems operate (Cameron et al., 2014). In recent years, this enhanced capability has contributed to an explosion of biotechnology research, affording engineers with greater control over cellular expression, and more precise instruments to engineer and nurture desired changes in the genome (Kozovska et al., 2021). The implications include potentially revolutionary treatments of debilitating disease, to environmental restoration, to various industrial advancements that address critical challenges in the future of global standards of living (El Karoui et al., 2019; Meng and Ellis, 2020; Cubillos-Ruiz et al., 2021). Other scholars have also explored options to make risk management measures more proportionate and adaptive to potential risks, uncertainties, and benefits (Devos et al., 2022).

Yet, despite 2 decades of improved understanding of synthetic biology and other emerging biotechnologies, uncertainty with respect to downstream implications (e.g., unintended exposure to novel genetic material, affecting human health and biodiversity alike) has grown rather than shrunk (Eriksson et al., 2020). This is due in part to the increased reach of biotechnology applications, including examples as species control (e.g., mosquito vectors for human pathogens—(Benelli et al., 2014), to de-extinction (e.g., wooly mammoth—McCauley et al., 2015), to biomining with engineered bacteria (Brune and Bayer, 2012), to the potential elimination of harmful heritable human diseases (Bosley et al., 2015) among many others. Many proposed biotechnology applications are intended for public or environmental release to maximize their beneficial potential, yet equally carry some measure of uncertain risks to proliferate in the environment or incur harms. In some ways, these risks are fundamentally unknowable up-front and require research and application to identify and characterize—creating a Catch-22 for risk averse risk cultures (Carter et al., 2014). And for instances where hazards have been identified, the research requirements to effectively bound risk in a manner consistent with many nations’ precautionary attitudes are prohibitive (Kuiken et al., 2014; Wareham and Nardini, 2015; Trump et al., 2018).

The result is a global regulatory environment forced to grapple with extreme uncertainty—including the possibility for global spillovers of biological risk events, however minute on a case-by-case basis. In-turn, such uncertainty limits the governance options of potential innovators: rather than a continuum of policy options that accounts for a rough bounding of technology risk against socioeconomic benefit, effective options are to (a) heavily restrict innovation to the point of no near-term market viability, or (b) permit near-free innovation potential, governed by existing capabilities for overall laboratory safety and material biosecurity (Lyall et al., 2012; Mandel, 2013; Lyall and Tait, 2019). Depending upon frames, perceived incentives, and local risk culture, both postures are individually rational despite being based upon near-identical starting points of uncertainty in risk and benefit.

Absent the pull towards a new set of international norms, values, or codes of conduct, individual nations will inevitably pursue biotechnology modernization platforms with less congruity to others over time. The implications of this include issues of safety (potential for accidents or unintended harmful consequences) and security (potential for deliberate misuse of biotechnology or its products). And, inevitably, this entrenchment will have decades of economic and health implications, where early actors may enjoy dominance over large swathes of a new field given their hard-won knowledge of translating elements of biotechnology into viable products. Likewise, however, exposure to potential novel hazards will be concentrated in aggressively developing nations, until safety protocols can be defined, regulated, and implemented. If successful, nations less willing to engage in early technology development risk becoming “captured” by the market capabilities of others (Wu et al., 2010; Adenle, 2011; Yrjola, et al., 2022). Subsequently, the divergence between a small number of early adopters and a larger body of risk averse nations will worsen the technology pacing problem, whereby the accelerating growth of innovation in the biotechnology space outstrips established best practices for environmental health and safety assessment as well as regulatory practices (Marchant et al., 2011; Fenwick et al., 2017; Trump et al., 2020). Closing this gap is no simple task—compelling *suis generis* hard law across the international landscape is doomed to clash against prevailing political and institutional debates and regulatory instruments. Scholars denote the possibility of soft law approaches as suggesting guidelines for best practice without compelling changes to national regulation, such as within the earliest days of genomics research at the Asilomar Conference on Recombinant DNA (Berg et al., 1975; Abels, 2005). Whether or not such a focusing event as a major conference can incentivize international commitment to biotechnology soft law after decades of national investment and regulatory development remains to be seen, and will likely be more complex, and more expensive in political and economic capital, than in the field’s early decades.

## 4 What does the next decade look like if such divergence is unchanged?

As the foundational life sciences of biotechnology continue to evolve and are increasingly integrated into product development, the task of governing biotechnology concurrently grows more intricate. The incorporation of these advanced scientific principles and techniques into the fabric of product design and development necessitates policies that comprehensively address not just the end products, but also the processes involved in their creation (NAS, 2018; Marris and Calvert, 2020). This expanded policy requirement contributes to an escalation in the complexity of strategic shifts, thus heightening the associated political and economic costs of switching technology policies. The inherent difficulty of navigating these changes serves to consolidate a natural preference for policy *status quo*, barring a significant crisis or catalytic event that necessitates a change (Sun et al., 2022). Consequently, the stakes are raised higher, with the cost of policy inertia becoming a significant factor in the wider discussion on the direction of biotechnology governance (Greer and Trump, 2019). This setting is how technology divergence forms and worsens, and has shaped the past 2 decades of international biotechnology research and development.

Early hints of what might become of the next ten to 20 years of international biotechnology competition are taking shape, though not guaranteed to transpire. Many capabilities are sought through biotechnology research, including those with iterative improvement over conventional product options (e.g., chemical production and synthesis), as well as revolutionary or groundbreaking (e.g., treatments or cures of debilitating diseases that current lack adequate interventions). While these application areas are numerous and growing with each year, they are generally summarized in government pronouncements as falling into various categories: biomanufacturing and biology-based fermentation of compounds, environmental deployment (e.g., environmental sensing and/or remediation by designed microbial systems against environmental targets), systems-enabled biotechnologies (broad-based genomic engineering to enable unique or novel phenotypic expression in a range of plants and animals), and bio-engineering for substances intended for ingestion, treatment, or gene therapy of humans (Titus et al., 2020).

Several governments are clarifying their biotechnology modernization strategies through public pronouncements, indicating the capabilities they seek to acquire. For example, in 2022, the Chinese National Development and Reform Commission (NDRC) shared the “14th Five-Year Plan for Bioeconomic Development” with a focus in synthetic biology in biomedicine, bio-agriculture, bio-manufacturing, and biosecurity, emphasizing China’s goal in achieving field leadership in medical and gene therapy breakthroughs for humans. In human subjects research, human genome editing was first reported in China in 2015 followed by a major study in 2017 that reported a successful correction of a defective gene in human embryos (Ma et al., 2017). This was followed by the starkly controversial claims of a Chinese scientist that used CRISPR embryonic genome of twins (Cyranoski, 2019; Science, 2023). The top-down policy approach can also be seen with government implementing legislation and regulation on biotechnology such as the 2022 Issues Guide for Bio-security Measurement of Gene Edited Crops (Reuters, 2022; Zhang et al., 2022) and the backing of 2020 Biosafety Law of the People’s Republic of China (Li et al., 2021). Local risk culture holds reservations on human subjects research, although more accepting attitudes towards research on germline gene editing that would reduce or eliminate heritability of debilitating disease (Zhang and Lie, 2018). While less centralized than with China, Japanese biotechnology development has engaged in ample research both upon countering human pathogens as well as research into transgenic food products in a more permissive regulatory environment than the United States or Europe (Fabbri et al., 2023). Other nations have less financially extensive biotechnology research enterprises—such as South Korea or Singapore—though have extensive research in government or university laboratories on furthering pharmaceuticals, medical interventions, or other benefits to human subjects (Mao et al., 2021).

Outside of human subjects research, other nations have established platforms to further production capacity of industrial enzymes, bio-engineered agriculture, and others. In 2019, the Russian Federation approved the “Federal Research Programme for Genetic Technologies Development for 2019–2027,” which stated that “the Programme’s key objectives are to implement a comprehensive solution to the task of the accelerated development of genetic technologies, including genetic editing; to establish scientific and technological ground for medicine, agriculture and industry; to improve the system of preventing biological emergencies and monitoring in this area.” Progress made within this program is expected to be carried out at new laboratories established at research and academic institutions, to increase biosecurity, and to ensure technological independence. It additionally aims to set up at least three world-class level centers for genome research, to design new lines of plants and animals, and to produce *in vitro* and *in vivo* models of human illnesses. Russia’s announcement in May of 2019 of a new $1.7 billion dollar program to promote the development of ten new varieties of gene-edited crops and animals by 2020 and twenty more by 2027 for a total of thirty in less than a decade demonstrates their commitment to the program, though lags behind others engaging with research on GMOs or chemical biosynthesis. This announcement suggests governmental exemption on the prohibition of the cultivation of GMOs in Russia (Dobrovidova, 2019).

The United States and European Union are longtime developers of genetic engineering and synthetic biology research, though with diverging regulatory traditions and risk cultures (Fabbri et al., 2023). Focusing on the process of biotechnology development (as opposed to product-focused regulation alone), EU governance has adopted risk averse interpretations of environmental, agricultural, and human subjects research, though more permissive of industrial development. Directives (such as 90/219/EEC on Contained Use of Genetically Modified Materials or 2001/18/EC on Deliberate Release into the Environment of Genetically Modified Materials, later amended by Directive 2018/350 which focused more squarely on environmental risk assessment) have served as a common approach to govern genetically modified organisms, where each member state is required to achieve identified Directive policy goals via their own means. For genetically modified organisms, this often includes the use of existing member state regulatory agencies to cover related research within the respective state’s political borders. This is driven by the sui generis framework for regulating biotechnology and genetically engineered organisms, which is comprised of the collection of Directives and Regulations that explicitly address requirements that govern the process and products of genetic engineering exercises. Specifically, Directives concerning the transfer of genes 2001/18/EC), the deliberate release of genetically modified microorganisms (90/220/EEC), the mutation and potential proliferation of genetically modified microorganisms and biodiversity impacts (2001/18/EC), laboratory and workplace safety with experiments conducting genetic modification (2009/41/EC and 2000/54/EC), general consumer health regulation for products with artificial genetic information (1829/2003), and specific Directives of pharmaceutical products containing artificial genetic material (726/2004) were viewed in literature as capable of covering existing iterations of “semi-synthetic” synthetic biology products, although may be challenged in the future as synthetic biologists are able to foster increasingly artificial synthetic biology -products such as with synthesized vaccines or other therapeutics. European regulation will eventually have to grapple with the question of how to govern fully synthetic cells which lack clear comparisons with products derived from naturally occurring components. Without an alternative to quantitative and comparative risk analysis between such products on a case-by-case basis, European regulatory protocols and requirements may hinder the further development and commercialization of potentially beneficial products as with new pharmaceuticals and vaccine components.

The sheer diversity of synthetic biology research in Europe presents EU regulators with a near impossible problem of trying to assess risk in many differing technological processes and product categories. In some areas, this impasse has spurred some (as with the European Union Court of Justice in a July 2018 ruling) to apply existing EU Directives from earlier generations of genetically modified organisms onto gene editing technologies like CRISPR, which may significantly slow progress on gene editing research in the European Union. In other areas like novel genomic techniques for food production, recent European Commission policy proposals may relax regulations on certain genetic techniques which may garner opportunities to circumvent barriers to market-entry in the future (European Commission, 2023).

Likewise, the United States has engaged in aggressive development in various areas of biotechnology research, though has encountered regulatory and ELSI hurdles in others. The US has been a major developer of engineered agriculture for decades, rising from less than 20% of planted soybean, cotton, and corn seeds in 1996 to over 90% by end-2018 (US Department of Agriculture, 2018). US governance of biotechnology has taken a more product-driven focus than the European Union, China, Japan, Russian Federation, or many other nations, with safety and security process measures captured within product-specific regulation via the Environmental Protection Agency (e.g., Toxic Substances Control Act), the Food and Drug Administration (e.g., Food, Drug, and Cosmetics Act), the US Department of Agriculture- Animal and Plant Health Inspection Service (APHIS) (e.g., Plant Pest Act) (Carter et al., 2014; Wang and Zhang, 2019). Likewise, agencies like APHIS and FDA are compelled to assess broad environmental impacts of products intended for environmental release, influencing permits and approvals, via the National Environmental Policy Act (NEPA). Updates to US hard law pertinent to emerging biotechnologies arise gradually—e.g., the Frank R. Lautenberg Chemical Safety for the 21st Century Act, which amended TSCA to bolster EPA funding for evaluation of existing and future chemical products and institute risk-based assessments of such substances (US Congress, 2016)- though such updates are slower than comparators in Europe and abroad (Trump, 2017). Likewise, US research into stem cells has encountered decades of political resistance and regulatory blocks for the past 2 decades relative to China or Japan, extending to germline editing research on human embryos (Fabbri et al., 2023).

The impact of the SARS-CoV-2 pandemic on international biotechnology funding and development cannot be overstated. Notably, the global crisis prompted a surge in public and private funding towards advancing vaccine research and development, diagnostics, and therapeutics, revealing the extraordinary potential of biotechnologies in addressing emergent health crises. Likewise, “policy windows” of institutional acceptance of certain biotechnologies opened, with a goal to address a rising hazard in the form of a novel human pathogen (Kingdon, 1993). Governments worldwide have recognized the critical role of biotechnology in protecting public health and have accordingly accelerated their investment into this domain. The pandemic has also demonstrated the potency of emerging biotechnologies like mRNA-based vaccines, exemplified by the Pfizer-BioNTech and Moderna COVID-19 vaccines, which were developed at a remarkable pace due to a combination of advanced biotechnological tools and substantial funding.

At the same time, the pandemic has necessitated a drastic shift in global collaborative efforts. Informal international data sharing arrangements were formed, and data was shared at an unprecedented scale to (a) evaluate the hazards and epidemiological trends of the SARS-CoV-2 virus and its variants, and (b) to enable the rapid development and distribution of vaccines (Corey et al., 2020; Cosgriff et al., 2020; Duan et al., 2022). This collective effort underscored the value of open and cooperative approaches to biotechnological advancement, although much of the informal international collaboration around health risk data analytics weakened as the pandemic progressed (Singh et al., 2021). However, the pandemic has also highlighted stark disparities between countries in their biotechnological capacities and their access to biotechnological solutions, such as COVID-19 vaccines (Lucas-Dominguez et al., 2021; Tatar et al., 2021). These disparities underscore the risk of a widening “biotech divide” between nations with robust biotechnology sectors and those without. This is a foretaste of future biotechnology divergence—whether it be a medical breakthrough or a cutting-edge economic capability.

As we consider the future trajectory of international biotechnology, one must consider the possibilities for early actor nations, such as China, to gain significant ground in areas of medical research and human subjects. With their top-down approach to biotechnology modernization and strategic focus on synthetic biology in biomedicine, China has the potential to significantly impact the global landscape in this regard. For instance, China has been notably aggressive in pursuing advancements in gene therapy and genome editing, as demonstrated by the first report of human genome editing in 2015 and the later controversial claims of CRISPR-edited human embryos. The relative permissiveness and adaptiveness of China’s regulatory environment, along with substantial state-backed funding for research, fosters an environment conducive to rapid advances and innovation. Moreover, the country’s strategic orientation and commitment to biotechnology as an essential driver of its national development agenda further propels its drive to attain leadership in these areas.

If China becomes a dominant player in the field of biotechnology for medicines and human health, the regulatory and economic implications would be substantial, both for China and the international community. Regulatory implications could include a shift towards the Chinese regulatory model. If China’s approach proves successful, it could influence international regulatory standards and norms for biotechnological products and practices. It may also prompt other nations to adjust their policies to remain competitive in the global biotech industry. The Chinese model, which is more product-based and with an emphasis on speed-to-market, could lead to a global acceleration in the development and approval of new treatments, but also raise questions around safety and ethical considerations. Economically, China’s dominance in biotechnology could have profound effects on global health markets. As a major producer of biotech products, China could potentially dictate pricing and distribution, influencing global health economics and accessibility to novel treatments. Furthermore, China’s dominance could shift the balance of trade, leading to a more East-centric global biotech economy. This may prompt Western companies to increase their investments in biotechnology to keep pace with China, fueling a global “biotech race” towards the longer-term, high-risk applications in human health and gene therapies.

Nations with large public-sector grants, as well as private-sector investment, will continue to excel in biotechnology innovation. The United States retains a dominant role here, including a vast university system and growing bioeconomy to sustain both a mature biotechnology workforce as well as a national economy with demands for biotechnology products. This will ensure competitiveness in most biotechnology development areas but does not guarantee leadership in all product applications. The United States is likely to retain field leadership over engineered agriculture due to a more permissive regulatory and consumer environment in that space, though will face substantial ELSI and regulatory hurdles to keep pace with other nations like China or Japan on human health applications.

Other nations may not achieve overall dominance across multiple biotechnology channels but can achieve leadership in a specific niche or product category. Some, like the Russian Federation or Pakistan, strive for mastery of biotechnology capabilities to facilitate industrial enzyme production and cash crop bioengineering, respectively (ISAAA, 2019). These targeted advances may allow them to keep pace with more well-funded developers like the United States or China and may even grant them some competitive advantage in niche applications of biotechnology for explicit products. Such niche leadership has been observed for other emerging technologies with significant economic and defense benefits—for example, both Estonia and Israel are lauded for their cybersecurity and digital security capabilities, despite having much smaller budgets and research enterprises than the United States, European Union, or Russian Federation (Herzog, 2017; Housen-Couriel, 2017).

Thus, without an international event or accord to align technology governance expectations and best practices, biotechnology divergence will foster an international landscape of clear leaders in specific technology areas, and a larger host of nations that are either (a) dependent upon the early adopter for desirable products, or (b) are locked out of the economic and defense benefits of those technologies. Likewise, early adopters have the privilege not only to set the market for product pricing, but also can exert considerable pressure upon their trade partners to align regulatory requirements around familiar, usually favorable terms (Zhang et al., 2020; Irwin, 2021). Such shifts in regulatory policy might be unpalatable or even impossible for some nations to embrace, depriving them of certain elements of the biotechnology market.

Biotechnology research is not guaranteed to be a fruitful endeavor for early adopters—many experiments will fail or be proven to be too risky to continue. For those that do succeed, however, biotechnology divergence will contribute to greater asymmetry amongst the global commons to understand, prevent, mitigate, govern, and communicate potentially novel hazards that biotechnology may incur to humans or the environment. While early actors incur greater exposure to these unique hazards (e.g., horizontal gene transfer), they also gain critical and usually proprietary or safeguarded knowledge critical to fostering effective safety and security norms and practices. As such, successful early adopters forge a path dependence in their biotechnology research that facilitates compounding improvement in knowledge and operability of biotechnology processes and products in a way that late-adopters will struggle to keep up with. In the coming decades, this gap in knowhow can leave late adopters less capable of governing biotechnology hazards that creep into their political borders, even despite moratoria (e.g., the spreading of animal pathogens or engineered seeds across political borders).

## 5 Discussion

Looking ahead, if the current divergence remains unmitigated, the implications are multifaceted. Biotechnology divergence has the potential to fundamentally reshape the geopolitical landscape, altering traditional power dynamics based on factors such as economic strength, military prowess, and natural resource availability. Early adopters of biotechnologies are likely to gain not only scientific and technological advantages but also significant diplomatic influence. By leading the development and implementation of new biotechnologies, these nations have the capacity to redefine global norms and standards and shape international policy in ways that protect their own interests and values. Furthermore, they can leverage their advanced capabilities to exert influence over other nations, whether through diplomacy, economic sanctions, or even technological coercion.

In addition, as early adopters establish themselves as central nodes in global biotechnology networks, they gain considerable economic advantages. Their prominence attracts investment, talent, and partnerships from around the world, fueling further innovation and bolstering their competitive position. In contrast, late adopters risk being sidelined in the global biotech industry. They may find themselves dependent on early adopters for access to vital biotech products and services, potentially facing higher costs and reduced availability. Additionally, their lagging capabilities may deter investment and talent, further widening the gap with early adopters.

The divergence in biotechnology capabilities and influence could exacerbate global inequities, fostering a world where access to the benefits of biotechnology—whether in health, agriculture, or industry—is unevenly distributed. This situation could lead to growing disparities in health outcomes, economic prosperity, and overall quality of life between nations. Furthermore, the concentration of power in a few early adopter nations might stifle global collaboration, hinder knowledge sharing, and create a more fragmented and competitive global biotech landscape.

Twenty years of research and billions of dollars of investment have commenced the process of biotechnology divergence. It is not guaranteed to continue, though absent a focusing event possessing significant harmful consequences to incentivize early adopters to internationally harmonize their technology modernization strategies, there is little incentive for early adopters to change their perceived prospects and sacrifice the potential economic, health, and defense rewards. Moreover, the clues of what that world might look like are unfolding and have considerable ramifications for the biotechnology marketplace of 2030 and beyond.

## Data Availability

The original contributions presented in the study are included in the article/Supplementary Material, further inquiries can be directed to the corresponding author.
